# Incidence of Carotid Blowout Syndrome in Patients with Head and Neck Cancer after Radiation Therapy: A Cohort Study

**DOI:** 10.3390/diagnostics14121222

**Published:** 2024-06-09

**Authors:** Jian-Lin Jiang, Joseph Tung-Chieh Chang, Chih-Hua Yeh, Ting-Yu Chang, Bing-Shen Huang, Pi-Shan Sung, Chien-Yu Lin, Kang-Hsing Fan, Yi-Chia Wei, Chi-Hung Liu

**Affiliations:** 1 Stroke Center and Department of Neurology, Chang Gung Memorial Hospital, Linkou Medical Center, Taoyuan 33333, Taiwan; 2 School of Medicine, College of Medicine, Chang Gung University, Taoyuan 33302, Taiwan; 3 Department of Radiation Oncology, Proton and Radiation Therapy Center, Chang Gung Medical Foundation, Linkou Chang Gung Memorial Hospital, Taoyuan 33333, Taiwan; 4 Taipei Chang Gung Head & Neck Oncology Group, Chang Gung Memorial Hospital, Linkou Medical Center, Taoyuan 33333, Taiwan; 5 Department of Neuroradiology, Chang Gung Memorial Hospital, Linkou Medical Center, Taoyuan 33333, Taiwan; 6 Department of Neurology, National Cheng Kung University Hospital, College of Medicine, National Cheng Kung University, Tainan 70101, Taiwan; 7 Radiation Research Core Laboratory, Chang Gung University, Chang Gung Memorial Hospital, Linkou Medical Center, Taoyuan 33333, Taiwan; 8 Department of Radiation Oncology, New Taipei Municipal Tu-Cheng Hospital, New Taipei City 236, Taiwan; 9 Department of Neurology, Keelung Chang Gung Memorial Hospital, Keelung 83301, Taiwan; 10 School of Traditional Chinese Medicine, College of Medicine, Chang Gung University, Taoyuan 33302, Taiwan; 11 Institute of Health Policy and Management, College of Public Health, National Taiwan University, Taipei 10055, Taiwan

**Keywords:** carotid blowout syndrome, head and neck cancer, nasopharyngeal carcinoma, pseudoaneurysm, radiation therapy

## Abstract

Carotid blowout syndrome (CBS) is a rare yet life-threatening complication that occurs after radiation therapy (RT). This study aimed to determine the incidence of CBS in patients with head and neck cancer (HNC) undergoing contemporary RT and to explore potential discrepancies in the risk of CBS between nasopharyngeal cancer (NPC) and non-NPC patients. A total of 1084 patients with HNC who underwent RT between 2013 and 2023 were included in the study. All patients were under regular follow-ups at the radio-oncology department, and underwent annual contrast-enhanced computed tomography and/or magnetic resonance imaging for cancer recurrence surveillance. Experienced neuroradiologists and vascular neurologists reviewed the recruited patients’ images. Patients were further referred to the neurology department for radiation vasculopathy evaluation. The primary outcome of this study was CBS. Patients were categorized into NPC and non-NPC groups and survival analysis was employed to compare the CBS risk between the two groups. A review of the literature on CBS incidence was also conducted. Among the enrolled patients, the incidence of CBS in the HNC, NPC, and non-NPC groups was 0.8%, 0.9%, and 0.7%, respectively. Kaplan–Meier analysis revealed no significant difference between the NPC and non-NPC groups (*p* = 0.34). Combining the findings for our cohort with those of previous studies revealed that the cumulative incidence of CBS in patients with HNC is 5% (95% CI = 3–7%) after both surgery and RT, 4% (95% CI = 2–6%) after surgery alone, and 5% (95% CI = 3–7%) after RT alone. Our findings indicate a low incidence of CBS in patients with HNC undergoing contemporary RT. Patients with NPC may have a CBS risk close to that of non-NPC patients. However, the low incidence of CBS could be a potentially cause of selection bias and underestimation bias.

## 1. Introduction

Head and neck cancer (HNC) is the seventh most common cancer worldwide, causing approximately 325,000 annual fatalities [1]. Radiation therapy (RT) is a crucial component of HNC treatment. Although the survival rates for HNC have considerably improved, managing the long-term complications related to RT for HNC has presented a new challenge. Carotid blowout syndrome (CBS) is a rare, yet life-threatening complication that occurs after RT. CBS is categorized into three types: (1) threatened CBS, involving exposure of the carotid artery due to wound breakdown or neoplastic invasion, leading to a risk of rupture if the artery is not promptly covered with healthy tissue; (2) impending, involving intermittent hemorrhage often originating from a ruptured carotid artery with a pseudoaneurysm; and (3) acute CBS, involving a severe and uncontrollable hemorrhage requiring immediate intervention that is beyond the management scope of surgical packing [2]. The mortality rate following a massive hemorrhage in acute CBS was reported to be as high as 75% [3].

Studies on CBS have predominantly employed case–control or cross-sectional designs. In the limited cohort studies that have been conducted, the incidence rate of CBS in patients with HNC following both surgical procedures and RT has been reported to range from 2.9% to 4.3% [4,5,6,7,8]. Notably, for individuals undergoing reirradiation (re-RT) due to recurrent HNC, the CBS incidence varies from 2.6% to 10% [9,10,11,12,13,14,15,16,17,18,19,20,21], with a median interval of 7.5 months between CBS occurrence and re-RT [2]. However, these findings were predominantly derived from the data related to HNC patients undergoing conventional RT. In addition, most of these studies were published before 2010. Advances in RT technology have gradually led to the radiation doses to normal tissues being reduced [22,23]. However, studies investigating whether contemporary RT is associated with a decrease in the risk of CBS are scarce. Addressing this question is crucial for patients and clinicians with respect to decision-making regarding the choice of RT method as well as that regarding follow-up plans after RT. Few studies have compared the incidence rate between different forms of HNC, although some have reported that patients with NPC may be more vulnerable to CBS development than those without. Patients with NPC may receive different treatment protocols, RT methods, and radiation field exposures when compared to non-NPC HNC patients [8,24]. However, the risk of CBS between NPC and non-NPC HNC patients remain uncertain. In this study, we reviewed the serial head and neck images of HNC patients who received contemporary RT methods including intensity modulation radiotherapy (IMRT), volume modulated modulation radiotherapy (VMAT), or intensity nodulation proton therapy (PBT) during their long-term follow-ups to determine the current incidence of CBS and compare the risk of CBS between different HNC types.

## 2. Material and Methods

### 2.1. Patient Recruitment and Data

**Figure 1:**
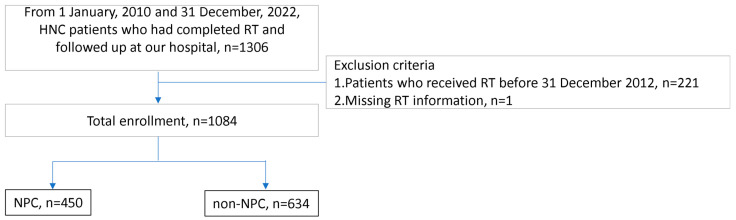
Flow of selection of study participants. Abbreviations: HNC, head and neck cancer; NPC, nasopharyngeal carcinoma; RT, radiation therapy.

In 2022, we initiated an am-bidirectional cohort study within our hospital (ClinicalTrials.gov identifier No.: NCT06111430). The study population comprised patients from our radio-oncology department who were either prospectively or retrospectively enrolled. A non-probability convenient sampling approach was employed for patient recruitment. Given the well-known risk of vasculopathy as a serious complication after RT, most patients readily consented to participate in this study after being thoroughly informed of its purpose. In the prospective cohort, we continually followed the patients after enrollment. In the retrospective cohort, we collected the clinical data of patients before the Ethics Institutional Review Board adoption date. The median retrospective period, calculated from the last RT date to the date of their enrollment in this study, was 35 months, whereas the median prospective follow-up duration after the date of enrollment in this study was 12 months. The first part of this cohort study focused on investigating the risk of CBS following contemporary RT; therefore, we included patients receiving IMRT, VMAT, and PBT only. We excluded patients received RT before 31 December 2012 because IMRT/VMAT and PBT were the major RT methods for HNC patients in our institution since 2013. Additionally, patients with missing RT information were excluded (Figure 1). The study protocol was approved by the Ethics and Institutional Review Board of our hospital (202101981B0, 202200464B0, and 202400107B0), and all eligible patients provided written informed consent. This study followed the Strengthening the Reporting of Observational Studies in Epidemiology reporting guidelines.

We systematically reviewed the demographic information and common risk factors for vascular disease of the recruited patients. These risk factors included dyslipidemia, hypertension, diabetes mellitus, and cigarette smoking [25]. Additionally, we collected laboratory data, including those on baseline glycated hemoglobin, low-density lipoprotein cholesterol, and serum creatinine levels. Furthermore, we obtained information regarding the types and stages of HNC, the total accumulated dose of RT, and the timing of the last RT fraction.

### 2.2. Grouping

The patients in this study were categorized into two groups based on cancer type: the NPC group and the non-NPC group. The non-NPC group comprised patients with oral cavity, oropharyngeal, laryngeal, or hypopharyngeal cancers (Figure 1).

### 2.3. Methods of RT

The treatment plans for PBT, IMRT, and VMAT were generated using the Eclipse planning system (version 13.7; Varian Medical Systems, Palo Alto, CA, USA). For PBT, worst-case robust optimization was used for clinical target volume coverage without planning target volume expansion but with set-up error and uncertainty in proton therapy planning. The planning target volume in IMRT/VMAT was expanded by 3–5 mm around the clinical target. The same dose constraints and optimization algorithm were applied for PBT and IMRT/VMAT. The relative biological equivalent value for PBT was assumed to be 1.1. The prescription comprised a dose of 6000–6600 cGy delivered in 30–33 fractions for postoperative radiotherapy and 6996 cGy delivered in 33 fractions for primary radiotherapy over 6–7 weeks (5 fractions per week). Simultaneous treatment of all target volumes was performed. Any total treatment time that exceeded the scheduled duration by more than 5 days was considered a major violation [26].

### 2.4. Follow-Up and Outcomes

The patients underwent regular follow-ups at the Department of Radiation Oncology at least once every 6 months. As part of their surveillance for cancer recurrence, head and neck computed tomography (CT) and/or magnetic resonance imaging (MRI) scans were conducted at 3 months after RT then then every 6 to 12 months if the last image reached clinical remission. For head and neck CT scans, a multidetector CT scanner was employed, enabling multiplanar reformation to generate thin-slice images (thickness, 3 mm) in the axial, coronal, and sagittal directions. MRI examinations of the head and neck were performed using a 1.5- or 3.0-Tesla MR scanner equipped with a standard head and neck coil. The MRI scans included precontrast T1-weighted images, T2-weighted fat-saturated images, and postcontrast T1-weighted fat-saturated images in the axial, coronal, and sagittal planes. The section thickness was 5 mm with a 2.5 mm intersection gap in the axial plane and 4 mm with a 1 mm gap in the sagittal and coronal planes. In instances where concerns arose regarding whether an image revealed CBS or a pseudoaneurysm, additional imaging modalities, such as CT angiography, MR angiography, and digital subtraction angiography, were arranged to provide a more in-depth assessment.

The primary outcome of this study was the occurrence of any form of CBS induced by RT. The arteries with bleeding and rupture directly invaded by the tumor were not included. The secondary outcomes were pseudoaneurysm and mortality. The outcomes were confirmed by an experienced neuroradiologist and two vascular neurologists through a thorough review of the images of the patients included in this study.

### 2.5. Statistical Analysis

All data were retrospectively analyzed using R version 4.3.1 (Beagle Scouts).

Kolmogorov–Smirnov tests were used to test continuous variables. Categorical variables, such as the presence of dyslipidemia, hypertension, diabetes mellitus, alcohol drinking, and cigarette smoking, were analyzed using the chi-square test, whereas continuous variables, including baseline glycated hemoglobin, low-density lipoprotein cholesterol, and serum creatinine levels, were assessed using the two-sample *t*-test (age) or Mann–Whitney test (radiation dose, following duration). Furthermore, the risk of CBS was compared between the NPC and non-NPC groups by using Kaplan–Meier analysis. Additionally, the number of patients with an advanced cancer stage was compared between the NPC and non-NPC groups through Kaplan–Meier analysis. A *p* value of <0.05 was considered to indicate significance.

To integrate and compare our study results with previously published data, a comprehensive literature review was conducted through PubMed and Medline. The keywords used for the literature search were “(head OR neck OR cervical*) AND (carotid blowout syndrome OR carotid pseudoaneurysm) AND (radiation* OR irradiation* OR radiotherapy*) AND stenosis”. The cumulative incidence of CBS, along with its corresponding 95% CI, was calculated and combined using a random-effects model in a meta-analysis. The I^2^ statistic was employed to evaluate the degree of heterogeneity. An I^2^ value of >50% indicated substantial heterogeneity. A forest plot was employed to visually represent the effect size observed in each study and the aggregated estimates.

## 3. Result

A total of 1306 patients were initially screened from the present am-directional cohort study. Of these patients, 221 had undergone RT before 31 December 2012 and were excluded. Additionally, one patient with missing RT information was excluded. Finally, 1084 patients with HNC were recruited (Figure 1). Among them, 450 (42%) were categorized into the NPC group, and 634 (58%) were categorized into the non-NPC group. In the non-NPC group, 448 (70.7%) had oral cancer, 98 (15.5%) had hypopharyngeal cancer, 62 (9.8%) had laryngeal cancer, and 26 (4%) had other types of HNC. The NPC group was younger (59 vs. 51; *p* < 0.001), had a lower male predominance (88% vs. 80%; *p* < 0.001), received a higher radiation dose (6600 vs. 6996 cGy; *p* < 0.001) as well as a higher frequency of chemotherapy (89% vs. 94%; *p* = 0.004), but had lower frequencies of current cigarette smoking (16% vs. 7%; *p* < 0.001) and undergoing surgical treatment (39% vs. 1.6%; *p* < 0.001). The frequency of re-RT was similar between the NPC and non-NPC groups (4.4% vs. 6.8%; *p* = 0.105). Additionally, no significant differences were observed between the two groups in terms of hypertension, diabetes mellitus, dyslipidemia, frequency of re-RT, and follow-up duration (Table 1). 

**Figure 2:**
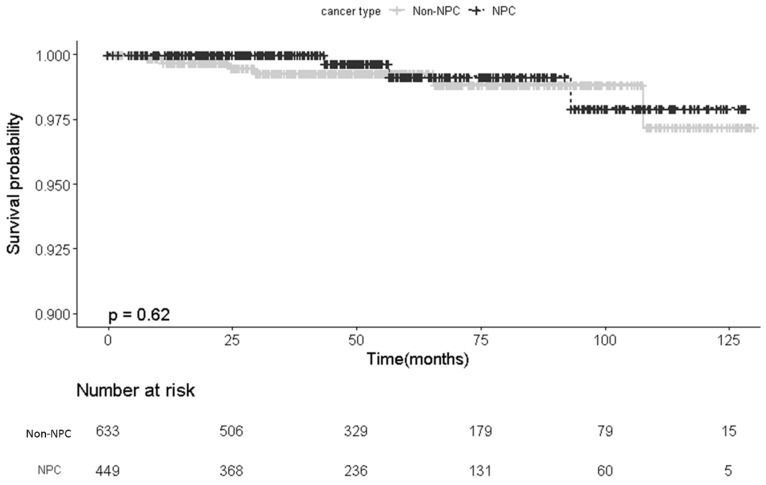
Results of Kaplan–Meier analysis of carotid blowout free rates between NPC and non-NPC groups after radiation therapy. The curves reveal a similar risk (*p* = 0.62) of a carotid blowout free event between the two groups. Abbreviation: NPC, nasopharyngeal carcinoma.

During the follow-up period, nine patients developed CBS, with six of these patients also exhibiting pseudoaneurysm. The overall incidence rate of CBS was 0.8% among the patients with HNC, with no significant difference in the incidence rate observed between the NPC and non-NPC groups (0.7% vs. 0.9%; *p* = 0.743). Additionally, the patients in the NPC group did not exhibit a higher proportion of pseudoaneurysm (0.7% vs. 0.5%, *p* = 0.697) or mortality (0% vs. 0.3%, *p* = 0.514) compared with the non-NPC group (Table 2). Subsequent survival analysis revealed no significant differences between the two groups (Figure 2). Of the two patients with CBS who died, one succumbed directly to CBS, whereas the other died due to infection after the CBS event (Table S1). 

**Figure 3:**
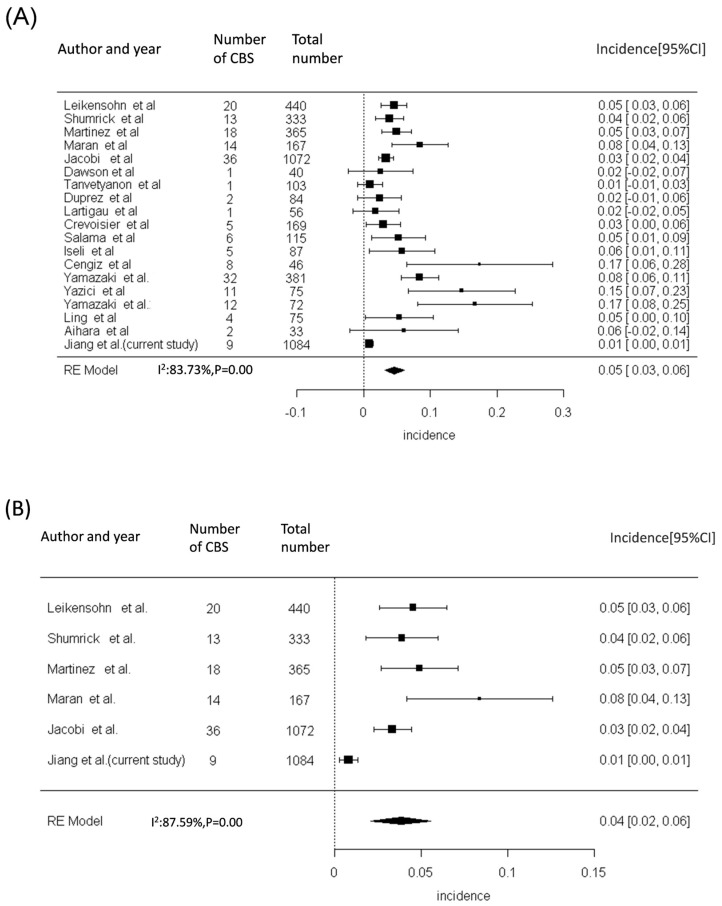

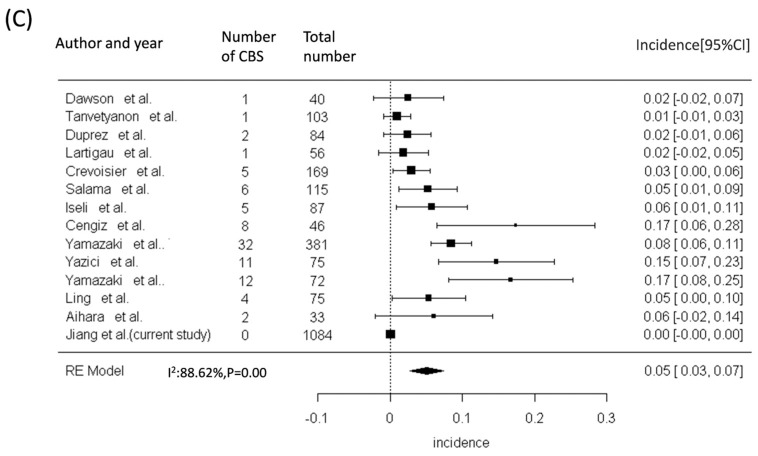
Cumulative incidence of CBS in patients with HNC after various treatments. The cumulative incidences of CBS were 5% (95% CI = 3-7%), 4% (95% CI = 2-6%), and 5% (95% CI = 3-7%) among patients with HNC undergoing both surgery and reirradiation therapy (**A**), surgery with or without radiation therapy (**B**), and reirradiation therapy alone (**C**), respectively. Abbreviations: CBS, carotid blowout syndrome; CI, confidence interval; HNC, head and neck cancer [4,5,6,7,8,9,10,11,12,13,14,15,16,17,18,19,20,21].

**Figure 4:**
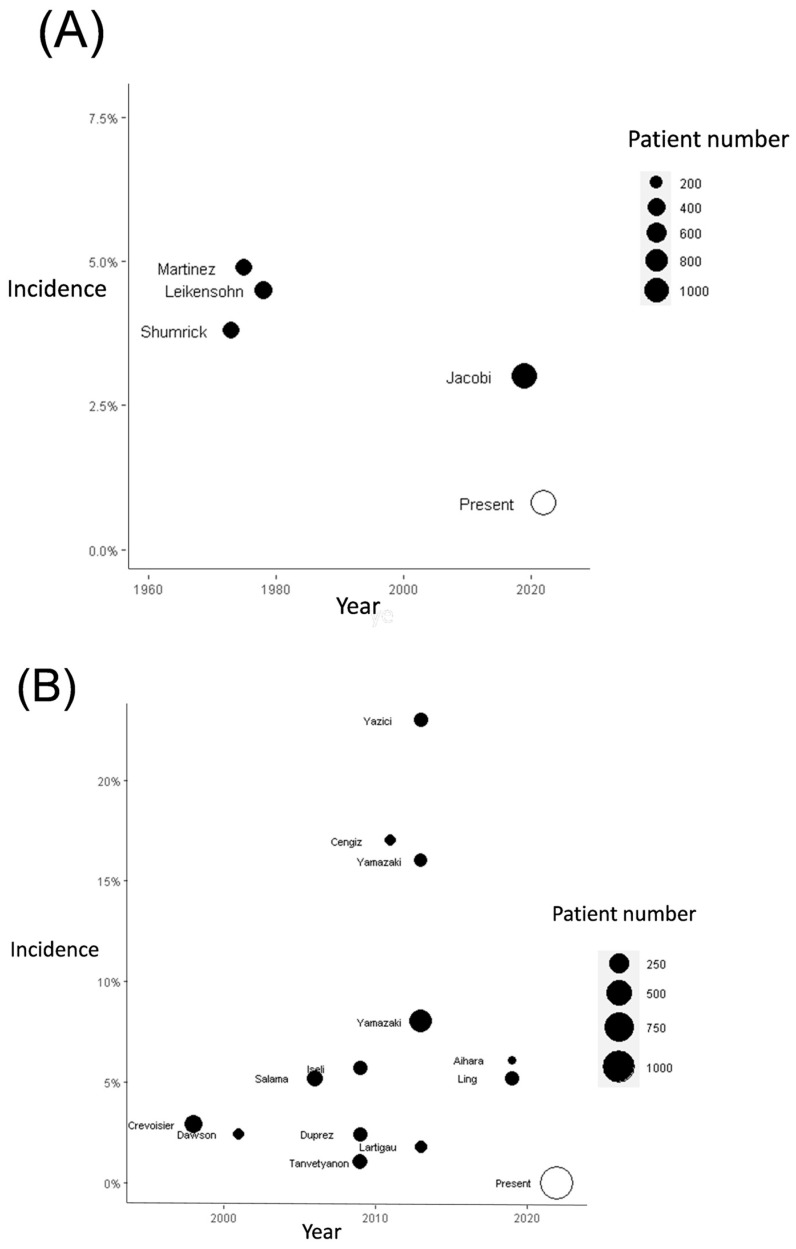
Summary of incidence of CBS in patients with HNC after (**A**) surgery and re-irradiation and (**B**) re-irradiation therapy based on findings from previous studies [4,5,6,7,8,9,10,11,12,13,14,15,16,17,18,19,20,21].

The incidence of CBS in the patients undergoing a combination of surgical treatment and re-RT has ranged from 3% to 8.4% in previous studies (Figure 3A and Figure 4A and Tables S2 and S3). Our meta-analysis, including the results of previous studies and our results, revealed a significantly higher risk of CBS in patients with HNC who have undergone both surgery and re-RT (Figure 3A), surgery with or without RT (Figure 3B), and re-RT alone (Figure 3C). The cumulative incidence of CBS was discovered to be 5% (95% CI = 3–7%), 4% (95% CI = 2–6%), and 5% (95% CI = 3–7%), respectively, in patients with HNC undergoing both surgery and re-RT, surgery with or without RT, and re-RT alone.

## 4. Discussion

Our cohort study revealed that the current incidence of CBS after RT is 0.8%, which is lower than that reported in previous studies [27,28,29,30]. Given the potential lethality of CBS and the perioperative cerebral ischemic rates observed in 4% to 14% of surviving patients after CBS [27,28,29,30], detailed discussions during the shared decision-making process and vascular screening for early CBS detection after RT may be required. Therefore, whether patients undergoing contemporary RT are associated with similar risks of CBS development should be addressed. 

The incidence rate of CBS in previous studies varied from 3% to 8.4% for patients undergoing both RT and surgical treatment. Most of these studies were retrospective and had small patient cohorts. The largest and latest retrospective study by Jacobi et al., involving 1072 patients with HNC who received RT between 2001 and 2011, reported a CBS incidence of 3% [8]. Our study predominantly enrolled patients who received RT after 2013 and discovered a lower incidence of CBS than that reported in Jacobi et al., which may be explained by advances in RT technology leading to a lower required radiation dose [22]. Notably, our study had a lower proportion of cases involving re-RT (5.8%) than that in a previous study (42.4%) [7]. The low frequency of re-RT in our study population may explain the lower CBS incidence in our data. Additionally, the definition of CBS in Jacobi et al. specifically included acute and life-threatening bleeding, and they did not investigate the frequency of pseudoaneurysm and impending CBS. The incidence of CBS in our study could be lowered to 0.2% after excluding pseudoaneurysm and impending CBS events. Our results contribute valuable information, filling a gap in the literature and providing noteworthy information for clinicians and patients.

A study reported a mortality rate of up to 40% in cases of CBS [31]. By contrast, the present study revealed a low mortality rate in patients with CBS (11.1%). This disparity indicates that early identification and intervention for pseudoaneurysm and impending CBS may contribute to a reduction in the occurrence of life-threatening events. Therefore, identifying high-risk groups susceptible to CBS development after RT is essential. The treatment methods, radiation doses, cancer staging, and radiation fields of patients with and without NPC with HNC considerably differ. Chemoradiation is the primary treatment for NPC [32], whereas surgery combined with chemoradiation is crucial for non-NPC HNC patients [33]; a study indicated that surgery and higher radiation doses to the neck may lead to damage in the carotid arteries [8]. A previous study showed that some chemotherapeutic agents, such as cisplatin and 5-Fluorouracil, may cause damage to endothelial cells and may induce local thrombosis [34]. A few reports disclosed inconclusive roles of oncological drugs in aneurysm growth [35,36]. However, our data were insufficient to demonstrate the casual relationship between chemotherapy and CBS occurrence. Despite the differences in the treatment for NPC and non-NPC cases, our study revealed a similar CBS incidence rate between the NPC and non-NPC groups.

Reports have identified risk factors for CBS other than the form of HNC, including re-RT, biopsy-proven pharyngeal/laryngeal chondronecrosis [22], and 360° involvement of the suspect artery by the tumor [37]. The meta-analysis in the current study revealed that the incidence of CBS after re-RT ranges from 1% to 23%, with an overall incidence of 5%. Cannavale et al. reviewed 24 patients with CBS who underwent CT angiography before CBS occurrence. They revealed that 360° involvement of the target vessel and advanced T stage increased the risk of CBS [37]. Feng et al. retrospectively reviewed 72 patients with HNC who received CT angiography before surgery and reported that 22 (30.6%) later developed CBS. They identified three independent risk factors, namely radical neck dissection, the carotid artery being surrounded by the tumor, and the carotid artery being invaded by the tumor without a clear boundary [38]. In the present study, of the nine patients who developed CBS, none had received re-RT, six had biopsy-proven pharyngeal and laryngeal chondronecrosis, and four had 360° involvement of the suspect artery by the tumor. However, the incidence of CBS was too low and may not be suitable for multivariate analyses. Additionally, our data did not reveal any influence of advanced cancer staging on CBS occurrence in either the NPC or the non-NPC group (Figure S1). Furthermore, the association between RT methods and CBS occurrence remains unclear. In the current study, the number of patients who received therapy was considerably lower and the follow-up period was considerably shorter for PBT than those for IMRT/VMAT. Furthermore, most patients who received PBT had NPC. Therefore, the current study was unable to draw conclusions regarding the influence of the RT method on CBS occurrence.

This study has several limitations. First, given the low incidence of CBS in our study, a larger sample size and a longer follow-up may be required in future studies to illustrate the differences more clearly in CBS risks among various RT methods and cancer types. Second, a study revealed that a radiation dose of over 70 Gy to the carotid artery could increase the risk of CBS [39]. However, we did not record the radiation dose to the carotid artery for the included patients, and therefore, we could not make assumptions regarding the relationship between radiation dose and CBS risk. Third, this was a single-institution study. The proportion of male NPC patients could be higher than previous studies [40]. Although our hospital is one of the largest HNC treatment centers and is the first medical center equipped to perform PBT in Taiwan. The generalizability of our conclusions to other populations may therefore be limited. Fourth, because not every patient in our institution was referred for enrollment in this study after RT, individuals who were lost to follow-up or died due to CBS may not be included in our data set. This potential selection bias may have led to an underestimation of the incidence of CBS in this study. Fifth, vascular status was evaluated through CT or MRI protocols designed for cancer follow-up, which may have affected the diagnosis of pseudoaneurysm. Although CT angiography was only arranged for some patients, this may have confounded the outcome assessment in this study. Despite these limitations, our study has several strengths. First, HNC is prevalent in the East Asian population, which may enhance the importance and generalizability of our study findings [41]. Second, our study represents one of the largest cohort studies addressing contemporary RT methods. The results may help mitigate concerns regarding the high CBS incidence in the current era.

## 5. Conclusions

Our cohort study revealed a lower incidence of CBS in patients with HNC undergoing contemporary RT than that reported in previous studies. Although the treatment approaches for NPC and non-NPC patients differ, our study revealed a similar risk of CBS between patients with these two cancer types. However, the low incidence of CBS could be a potentially cause of selection bias and underestimation bias. A multicenter prospective study is warranted to address several uncertainties regarding this risk.

## Figures and Tables

**Table 1:** Baseline characteristic of patients in NPC and non-NPC groups.

	Total, *n* = 1084	Non-NPC, *n* = 634	NPC, *n* = 450	*p*-Value
Gender, male (%)	915 (84%)	555 (88%)	360 (80%)	<0.001
Age, years	56 (48, 63)	59 (51, 65)	51 (43, 59)	<0.001
Diabetes mellitus (%)	123 (11%)	76 (12%)	47 (10%)	0.430
Hypertension (%)	198 (18%)	127 (20%)	71 (16%)	0.074
Dyslipidemia (%)	150 (14%)	90 (14%)	60 (13%)	0.685
Smoking				<0.001
Never	619 (57%)	306 (48%)	313 (70%)	
Current	137 (13%)	102 (16%)	35 (7%)	
Former	328 (30%)	226 (36%)	102 (23%)	
Cancer type				<0.001
NPC	450(41.5%)	0	450(100%)	
Oral cancer	448(41.3%)	448(70.7%)	0	
Hypopharyngeal cancer	98(9%)	98(15.5%)	0	
Laryngeal cancer	62(5.7%)	62(9.8%)	0	
Others	26(2.5%)	26(4%)	0	
Proton therapy (%)	241 (22%)	82 (13%)	159 (35%)	<0.001
Reirradiation (%)	63 (5.8%)	43 (6.8%)	20 (4.4%)	0.105
Radiation dose (Mean ± standard deviation, Centi-gray)	6996 ± 749	6600 ± 692	6996 ± 785	<0.001
Chemotherapy	992 (92%)	567 (89%)	425 (94%)	0.004
Surgery (%)	271 (25%)	246 (39%)	7 (1.6%)	<0.001
Follow-up duration, month	52.3 (30.3, 80)	52 (29.8, 80.5)	52.5 (30.75, 79.3)	0.711

*n* (%); Median (IQR). Abbreviations: NPC, nasopharyngeal carcinoma.

**Table 2:** Primary and secondary outcomes of this study.

Characteristic	Total, *n* = 1084	Non-NPC, *n* = 634	NPC, *n* = 450	*p*-Value
**Primary outcome**				
Carotid blowout syndrome	9 (0.8%)	6 (0.9%)	3 (0.7%)	0.743
**Secondary outcomes**				
Pseudoaneurysm	6 (0.6%)	3 (0.5%)	3(0.7%)	0.697
Mortality	2 (0.2%)	2 (0.3%)	0 (0%)	0.514

Abbreviation: NPC: nasopharyngeal carcinoma.

## Data Availability

The data sets generated during this study are available from the corresponding author upon reasonable request.

## Supplementary Materials

The following supporting information can be downloaded at: https://www.mdpi.com/article/10.3390/diagnostics14121222/s1, Figure S1: Survival analysis of CBS free rate between advanced and low cancer stages in NPC (A) and non-NPC (B) groups after radiation therapy. Table S1: Characteristics of nine patients with CBS. Table S2: Summary of studies on CBS in patients with HNC after surgery and radiation therapy. Table S3: Summary of previous studies regarding CBS in HNC patients after reirradiation therapy. References [4,5,6,7,8,9,10,11,12,13,14,15,16,17,18,19,20,21] are cited in the Supplementary Materials.

## Abbreviations

CBS: Carotid blowout syndrome; CT: Computed tomography; HNC: Head and neck cancer; IMRT: Intensity modulation radiotherapy; MRI: Magnetic resonance imaging; NPC: Nasopharyngeal cancer; PBT: Intensity nodulation proton therapy; RT: Radiation therapy; VMAT: Volume modulated modulation radiotherapy

## References

[1] Sung H., Ferlay J., Siegel R.L., Laversanne M., Soerjomataram I., Jemal A., Bray F. (2021). Global Cancer Statistics 2020: GLOBOCAN Estimates of Incidence and Mortality Worldwide for 36 Cancers in 185 Countries. CA Cancer J. Clin..

[2] Suarez C., Fernandez-Alvarez V., Hamoir M., Mendenhall W.M., Strojan P., Quer M., Silver C.E., Rodrigo J.P., Rinaldo A., Ferlito A. (2018). Carotid blowout syndrome: Modern trends in management. Cancer Manag. Res..

[3] Chang F.C., Lirng J.F., Luo C.B., Guo W.Y., Teng M.M., Tai S.K., Chang C.Y. (2007). Carotid blowout syndrome in patients with head-and-neck cancers: Reconstructive management by self-expandable stent-grafts. AJNR Am. J. Neuroradiol..

[4] Leikensohn J., Milko D., Cotton R. (1978). Carotid artery rupture. Management and prevention of delayed neurologic sequelae with low-dose heparin. Arch. Otolaryngol..

[5] Shumrick D.A. (1973). Carotid artery rupture. Laryngoscope.

[6] Martinez S.A., Oller D.W., Gee W., deFries H.O. (1975). Elective carotid artery resection. Arch. Otolaryngol..

[7] Maran A.G., Amin M., Wilson J.A. (1989). Radical neck dissection: A 19-year experience. J. Laryngol. Otol..

[8] Jacobi C., Gahleitner C., Bier H., Knopf A. (2019). Chemoradiation and local recurrence of head and neck squamous cell carcinoma and the risk of carotid artery blowout. Head Neck.

[9] Dawson L.A., Myers L.L., Bradford C.R., Chepeha D.B., Hogikyan N.D., Teknos T.N., Terrell J.E., Wolf G.T., Eisbruch A. (2001). Conformal re-irradiation of recurrent and new primary head-and-neck cancer. Int. J. Radiat. Oncol. Biol. Phys..

[10] Tanvetyanon T., Padhya T., McCaffrey J., Zhu W., Boulware D., Deconti R., Trotti A. (2009). Prognostic factors for survival after salvage reirradiation of head and neck cancer. J. Clin. Oncol..

[11] Duprez F., Madani I., Bonte K., Boterberg T., Vakaet L., Derie C., De Gersem W., De Neve W. (2009). Intensity-modulated radiotherapy for recurrent and second primary head and neck cancer in previously irradiated territory. Radiother. Oncol..

[12] Lartigau E.F., Tresch E., Thariat J., Graff P., Coche-Dequeant B., Benezery K., Schiappacasse L., Degardin M., Bondiau P.Y., Peiffert D. (2013). Multi institutional phase II study of concomitant stereotactic reirradiation and cetuximab for recurrent head and neck cancer. Radiother. Oncol..

[13] De Crevoisier R., Bourhis J., Domenge C., Wibault P., Koscielny S., Lusinchi A., Mamelle G., Janot F., Julieron M., Leridant A.M. (1998). Full-dose reirradiation for unresectable head and neck carcinoma: Experience at the Gustave-Roussy Institute in a series of 169 patients. J. Clin. Oncol..

[14] Salama J.K., Vokes E.E., Chmura S.J., Milano M.T., Kao J., Stenson K.M., Witt M.E., Haraf D.J. (2006). Long-term outcome of concurrent chemotherapy and reirradiation for recurrent and second primary head-and-neck squamous cell carcinoma. Int. J. Radiat. Oncol. Biol. Phys..

[15] Iseli T.A., Iseli C.E., Rosenthal E.L., Caudell J.J., Spencer S.A., Magnuson J.S., Smith A.N., Carroll W.R. (2009). Postoperative reirradiation for mucosal head and neck squamous cell carcinomas. Arch. Otolaryngol. Head Neck Surg..

[16] Cengiz M., Özyiğit G., Yazici G., Doğan A., Yildiz F., Zorlu F., Gürkaynak M., Gullu I.H., Hosal S., Akyol F. (2011). Salvage reirradiaton with stereotactic body radiotherapy for locally recurrent head-and-neck tumors. Int. J. Radiat. Oncol. Biol. Phys..

[17] Yamazaki H., Ogita M., Kodani N., Nakamura S., Inoue H., Himei K., Kotsuma T., Yoshida K., Yoshioka Y., Yamashita K. (2013). Frequency, outcome and prognostic factors of carotid blowout syndrome after hypofractionated re-irradiation of head and neck cancer using CyberKnife: A multi-institutional study. Radiother. Oncol..

[18] Yazici G., Sanlı T.Y., Cengiz M., Yuce D., Gultekin M., Hurmuz P., Yıldız F., Zorlu F., Akyol F., Gurkaynak M. (2013). A simple strategy to decrease fatal carotid blowout syndrome after stereotactic body reirradiaton for recurrent head and neck cancers. Radiat. Oncol..

[19] Karam I., Yao M., Heron D.E., Poon I., Koyfman S.A., Yom S.S., Siddiqui F., Lartigau E., Cengiz M., Yamazaki H. (2017). Survey of current practices from the International Stereotactic Body Radiotherapy Consortium (ISBRTC) for head and neck cancers. Future Oncol..

[20] Ling D.C., Vargo J.A., Gebhardt B.J., Grimm R.J., Clump D.A., Ferris R.L., Ohr J.P., Heron D.E. (2019). Dose-response modeling the risk of carotid bleeding events after stereotactic body radiation therapy for previously irradiated head and neck cancer. J. Radiosurg SBRT.

[21] Aihara T., Hiratsuka J., Ishikawa H., Kumada H., Ohnishi K., Kamitani N., Suzuki M., Sakurai H., Harada T. (2015). Fatal carotid blowout syndrome after BNCT for head and neck cancers. Appl. Radiat. Isot..

[22] Padua P.F., Fang H.Y., Young C.K., Yeh C.H., Lin C.C., Liao C.T., Chang T.J., Tsao C.K., Huang S.F. (2022). Carotid arterial blowout after organ preserving chemoradiation therapy in hypopharyngeal cancer. Medicine.

[23] Hall W.A., Paulson E., Li X.A., Erickson B., Schultz C., Tree A., Awan M., Low D.A., McDonald B.A., Salzillo T. (2022). Magnetic resonance linear accelerator technology and adaptive radiation therapy: An overview for clinicians. CA Cancer J. Clin..

[24] Lu H.J., Chen K.W., Chen M.H., Chu P.Y., Tai S.K., Wang L.W., Chang P.M., Yang M.H. (2013). Predisposing factors, management, and prognostic evaluation of acute carotid blowout syndrome. J. Vasc. Surg..

[25] Brown R.D., Broderick J.P. (2014). Unruptured intracranial aneurysms: Epidemiology, natural history, management options, and familial screening. Lancet Neurol..

[26] Wang H.M., Lin C.Y., Hsieh C.H., Hsu C.L., Fan K.H., Chang J.T., Huang S.F., Kang C.J., Liao C.T., Ng S.H. (2017). Induction chemotherapy with dose-modified docetaxel, cisplatin, and 5-fluorouracil in Asian patients with borderline resectable or unresectable head and neck cancer. J. Formos. Med. Assoc..

[27] Weinberg J.H., Sweid A., Joffe D., Piper K., Abbas R., Hussain Z., Anderson B., Gooch M.R., Herial N., Tjoumakaris S. (2020). Carotid Blowout Management in the Endovascular Era. World Neurosurg..

[28] Manzoor N.F., Rezaee R.P., Ray A., Wick C.C., Blackham K., Stepnick D., Lavertu P., Zender C.A. (2017). Contemporary management of carotid blowout syndrome utilizing endovascular techniques. Laryngoscope.

[29] Liang N.L., Guedes B.D., Duvvuri U., Singh M.J., Chaer R.A., Makaroun M.S., Sachdev U. (2016). Outcomes of interventions for carotid blowout syndrome in patients with head and neck cancer. J. Vasc. Surg..

[30] Chang F.C., Luo C.B., Lirng J.F., Lin C.J., Lee H.J., Wu C.C., Hung S.C., Guo W.Y. (2015). Endovascular Management of Post-Irradiated Carotid Blowout Syndrome. PLoS ONE.

[31] Bond K.M., Brinjikji W., Murad M.H., Cloft H.J., Lanzino G. (2017). Endovascular treatment of carotid blowout syndrome. J. Vasc. Surg..

[32] Rueda Domínguez A., Cirauqui B., García Castaño A., Alvarez Cabellos R., Carral Maseda A., Castelo Fernández B., Iglesias Rey L., Rubió-Casadevall J., Arrazubi V., Mesía R. (2022). SEOM-TTCC clinical guideline in nasopharynx cancer (2021). Clin. Transl. Oncol..

[33] Board P.D.Q.A.T.E. (2002). Laryngeal Cancer Treatment (PDQ^®^): Health Professional Version. PDQ Cancer Information Summaries.

[34] Herrmann J., Yang E.H., Iliescu C.A., Cilingiroglu M., Charitakis K., Hakeem A., Toutouzas K., Leesar M.A., Grines C.L., Marmagkiolis K. (2016). Vascular Toxicities of Cancer Therapies: The Old and the New--An Evolving Avenue. Circulation.

[35] Mikus E., Zucchetta F., Carigi S., Tripodi A. (2021). Asymptomatic giant circumflex aneurysm in patient with a cisplatin treatment history. J. Card. Surg..

[36] Leopardi M., Di Marco E., Musilli A., Ricevuto E., Bruera G., Ventura M. (2017). Effects of Chemotherapy in Patients with Concomitant Aortic Aneurysm and Malignant Disease. Ann. Vasc. Surg..

[37] Cannavale A., Corona M., Nardis P., De Rubeis G., Cannavale G., Santoni M., De Gyurgyokai S.Z., Catalano C., Bezzi M. (2020). Computed Tomography Angiography findings can predict massive bleeding in head and neck tumours. Eur. J. Radiol..

[38] Feng K., Hu J., Huang Q., Cai W., Zhuang Z., Liu H., Hou J., Liu X., Wang C. (2022). Risk factors and nomogram for predicting carotid blowout syndrome based on computed tomography angiography. Oral. Dis..

[39] Chen Y.J., Wang C.P., Wang C.C., Jiang R.S., Lin J.C., Liu S.A. (2015). Carotid blowout in patients with head and neck cancer: Associated factors and treatment outcomes. Head Neck.

[40] Yang T.H., Xirasagar S., Cheng Y.F., Chen C.S., Chang W.P., Lin H.C. (2023). Trends in the incidence of head and neck cancer: A nationwide population-based study. Oral. Oncol..

[41] Yamazaki H., Suzuki G., Aibe N., Nakamura S., Yoshida K. (2021). Fractionation or tumor factors-what matters in carotid blowout syndrome?. Strahlenther. Onkol..

