# Supplementary material for: Incidence of Carotid Blowout Syndrome in Patients with Head and Neck Cancer after Radiation Therapy: A Cohort Study
Source: Diagnostics (Basel). 2024 Jun 9;14(12):1222. doi: 10.3390/diagnostics14121222 (PMC11202696; doi:10.3390/diagnostics14121222)
Supplement: Supplementary file 1 [file diagnostics-14-01222-s001.zip › diagnostics-3025815-supplementary.pdf]

Supplementary materials

S1 figure. Survival analysis of CBS free rate between advanced and low cancer stages in NPC (A) and non-NPC (B) groups after radiation therapy.

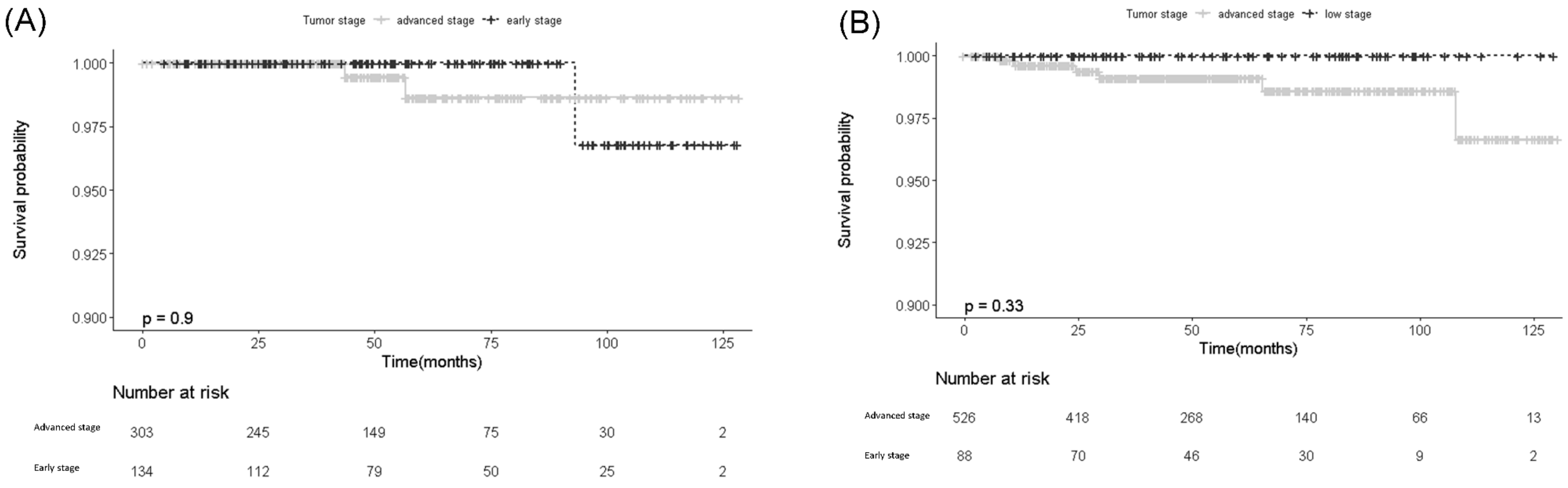

Abbreviations: CBS, carotid blow out syndrome; NPC, nasopharyngeal carcinoma.

S1 table. Characteristics of nine patients with CBS

	**Age**	**Gender**	**Location**	**Pseudoaneurysm**	**Cancer type**	**mortality**	**Reason of mortality**
1	54	Male	Right ICA	-	Hypopharyngeal cancer	-	NA
2	43	Male	Left ICA	+	NPC	-	NA
3	56	Male	Left ICA	+	NPC	-	NA
4	45	Male	Right ICA	+	Hypopharyngeal cancer	-	NA
5	54	Female	Left ICA	+	Left tonsil cancer	-	NA
6	54	Female	Right ICA	-	Hypopharyngeal cancer	+	Sepsis
7	65	Male	Right CCA	+	Hypopharyngeal cancer	+	CBS
8	56	Male	Left ICA	+	NPC	-	NA
9	70	Male	Left ECA	-	Right upper gum cancer	-	NA

Abbreviations: CBS, carotid blowout syndrome; CCA, common carotid artery; ECA, external carotid artery; ICA: internal carotid artery; NPC, nasopharyngeal carcinoma; NA, not applicable.

S2 table. Summary of studies on CBS in patients with HNC after surgery and radiation therapy.

Author	Duration	Patients (n)	CBS (n)	Median follow-up (month)	Study design	Radiation type	Reirradiation	Incidence
Leikensohn et al. [1]	1969-1973	440	20	NA	Retrospective	NA	NA	4.5%
Shumrick et al. [2]	1969-1972	333	13	NA	Retrospective	NA	NA	3.8%
Martinez et al.[3]	1964-1974	365	18	NA	Retrospective	NA	NA	4.9%
Maran et al.[4]	1969-1986	167	14	NA	Retrospective	NA	42.4%	8.4%
Jacobi et al.[5]	2001-2011	1072	36	14	Retrospective	Ch/Rt	NA	3%
Jiang et al. (Current study)	2013-2022	1084	9	17	Prospective	VMAT/PBT	5.8%	0.8%

Abbreviations: CBS: carotid blowout syndrome, Ch/Rt: chemotherapy/radiation therapy, HNC, head and neck cancer; NA, not available; VMAT: volumetric modulated arc therapy, PBT: proton therapy.

S3 table. Summary of previous studies regarding CBS in HNC patients after reirradiation therapy.

**Author**	**Duration**	**Patients (n)**	**CBS (n)**	**Median follow-up (month)**	**Study design**	**Radiation type**	**Incidence**
Dawson et al.[6]	1983-1999	40	1	12.5	Prospective	3D-CRT	2.5%
Tanvetyanon et al.[7]	1998-2008	103	1	19.3	Retrospective	Various ± Ch	1%
Duprez et al.[8]	1997-2008	84	2	49.5	Retrospective	IMRT	2.4%
Lartigau et al.[9]	2007-2010	56	1	38	Prospective	SBRT	1.8%
Crevoisier et al.[10]	1980-1996	169	5	33	Retrospective	Ch/Rt	2.9%
Salama et al.[11]	199-2004	115	6	67.4	Prospective	Ch/Rt	5.2%
Iseli et al.[12]	1992-2007	87	5	17.3	Retrospective	Ch/Rt	5.7%
Cengiz et al.[13]	2007-2009	46	8	11.93	Retrospective	SBRT	17%
Yamazaki et al.[14]	2000-2010	381	32	5	Prospective	SBRT	8%
Yazici et al.[15]	2007-2011	75	11	23	Prospective	SBRT	23%
Yamazaki et al.[16]	2000-2010	72	12	5	Prospective	SBRT	16%
Ling et al.[17]	2008-2013	75	4	37	Retrospective	SBRT	5.3%
Aihara et al.[18]	2003-2011	33	2	-	Retrospective	Boron neutron capture therapy	6.1%

Abbrev. CBS: Carotid blowout syndrome, Ch/Rt: chemotherapy/ radiation therapy, IMRT: intensity modulated radiation therapy, PT: proton therapy, SBRT: stereotactic body radiation therapy, VMAT: volumetric modulated arc therapy, 3D-CRT, three-dimensional conformal radiation therapy

Reference:

1. Leikensohn, J.;D. Milko, and R. Cotton, *Carotid artery rupture. Management and prevention of delayed neurologic sequelae with low-dose heparin.* Arch Otolaryngol, 1978. **104**(6): p. 307-10,https://doi.org/10.1001/archotol.1978.00790060009002
2. Shumrick, D.A., *Carotid artery rupture.* Laryngoscope, 1973. **83**(7): p. 1051-61,https://doi.org/10.1288/00005537-197307000-00006
3. Martinez, S.A.;D.W. Oller;W. Gee, and H.O. deFries, *Elective carotid artery resection.* Arch Otolaryngol, 1975. **101**(12): p. 744-7,https://doi.org/10.1001/archotol.1975.00780410036008
4. Maran, A.G.;M. Amin, and J.A. Wilson, *Radical neck dissection: a 19-year experience.* J Laryngol Otol, 1989. **103**(8): p. 760-4,https://doi.org/10.1017/s002221510011000x
5. Jacobi, C.;C. Gahleitner;H. Bier, and A. Knopf, *Chemoradiation and local recurrence of head and neck squamous cell carcinoma and the risk of carotid artery blowout.* Head Neck, 2019. **41**(9): p. 3073-3079,https://doi.org/10.1002/hed.25796
6. Dawson, L.A.;L.L. Myers;C.R. Bradford;D.B. Chepeha;N.D. Hogikyan;T.N. Teknos, . . . A. Eisbruch, *Conformal re-irradiation of recurrent and new primary head-and-neck cancer.* Int J Radiat Oncol Biol Phys, 2001. **50**(2): p. 377-85,https://doi.org/10.1016/s0360-3016(01)01456-0
7. Tanvetyanon, T.;T. Padhya;J. McCaffrey;W. Zhu;D. Boulware;R. Deconti, and A. Trotti, *Prognostic factors for survival after salvage reirradiation of head and neck cancer.* J Clin Oncol, 2009. **27**(12): p. 1983-91,https://doi.org/10.1200/jco.2008.20.0691
8. Duprez, F.;I. Madani;K. Bonte;T. Boterberg;L. Vakaet;C. Derie, . . . W. De Neve, *Intensity-modulated radiotherapy for recurrent and second primary head and neck cancer in previously irradiated territory.* Radiother Oncol, 2009. **93**(3): p. 563-9,https://doi.org/10.1016/j.radonc.2009.10.012
9. Lartigau, E.F.;E. Tresch;J. Thariat;P. Graff;B. Coche-Dequeant;K. Benezery, . . . A. Kramar, *Multi institutional phase II study of concomitant stereotactic reirradiation and cetuximab for recurrent head and neck cancer.* Radiother Oncol, 2013. **109**(2): p. 281-5,https://doi.org/10.1016/j.radonc.2013.08.012
10. De Crevoisier, R.;J. Bourhis;C. Domenge;P. Wibault;S. Koscielny;A. Lusinchi, . . . F. Eschwege, *Full-dose reirradiation for unresectable head and neck carcinoma: experience at the Gustave-Roussy Institute in a series of 169 patients.* J Clin Oncol, 1998. **16**(11): p. 3556-

62,https://doi.org/10.1200/jco.1998.16.11.3556
11. Salama, J.K.;E.E. Vokes;S.J. Chmura;M.T. Milano;J. Kao;K.M. Stenson, . . . D.J. Haraf, *Long-term outcome of concurrent chemotherapy and reirradiation for recurrent and second primary head-and-neck squamous cell carcinoma.* Int J Radiat Oncol Biol Phys, 2006. **64**(2): p. 382-91,https://doi.org/10.1016/j.ijrobp.2005.07.005
12. Iseli, T.A.;C.E. Iseli;E.L. Rosenthal;J.J. Caudell;S.A. Spencer;J.S. Magnuson, . . . W.R. Carroll, *Postoperative reirradiation for mucosal head and neck squamous cell carcinomas.* Arch Otolaryngol Head Neck Surg, 2009. **135**(11): p. 1158-64,https://doi.org/10.1001/archoto.2009.161
13. Cengiz, M.;G. Özyiğit;G. Yazici;A. Doğan;F. Yildiz;F. Zorlu, . . . F. Akyol, *Salvage reirradiaton with stereotactic body radiotherapy for locally recurrent head-and-neck tumors.* Int J Radiat Oncol Biol Phys, 2011. **81**(1): p. 104-9,https://doi.org/10.1016/j.ijrobp.2010.04.027
14. Yamazaki, H.;M. Ogita;N. Kodani;S. Nakamura;H. Inoue;K. Himei, . . . H. Udono, *Frequency, outcome and prognostic factors of carotid blowout syndrome after hypofractionated re-irradiation of head and neck cancer using CyberKnife: a multi-institutional study.* Radiother Oncol, 2013. **107**(3): p. 305-9,https://doi.org/10.1016/j.radonc.2013.05.005
15. Yazici, G.;T.Y. Sanlı;M. Cengiz;D. Yuce;M. Gultekin;P. Hurmuz, . . . G. Ozyigit, *A simple strategy to decrease fatal carotid blowout syndrome after stereotactic body reirradiaton for recurrent head and neck cancers.* Radiat Oncol, 2013. **8**: p. 242,https://doi.org/10.1186/1748-717x-8-242
16. Yamazaki, H.;G. Suzuki;N. Aibe;S. Nakamura, and K. Yoshida, *Fractionation or tumor factors-what matters in carotid blowout syndrome?* Strahlenther Onkol, 2021. **197**(8): p. 744-745,https://doi.org/10.1007/s00066-021-01767-4
17. Ling, D.C.;J.A. Vargo;B.J. Gebhardt;R.J. Grimm;D.A. Clump;R.L. Ferris, . . . D.E. Heron, *Dose-response modeling the risk of carotid bleeding events after stereotactic body radiation therapy for previously irradiated head and neck cancer.* J Radiosurg SBRT, 2019. **6**(2): p. 83-89
18. Aihara, T.;J. Hiratsuka;H. Ishikawa;H. Kumada;K. Ohnishi;N. Kamitani, . . . T. Harada, *Fatal carotid blowout syndrome after BNCT for head and neck cancers.* Appl Radiat Isot, 2015. **106**: p. 202-6,https://doi.org/10.1016/j.apradiso.2015.08.007
